# A Simple Sign for Recognizing Off–Axis OCT Measurement Beam Placement in the Context of Multicentre Studies

**DOI:** 10.1371/journal.pone.0048222

**Published:** 2012-11-08

**Authors:** Lisanne J. Balk, Willemien A. E. J. de Vries–Knoppert, Axel Petzold

**Affiliations:** 1 MS Centre Amsterdam, VU University Medical Centre, Amsterdam, The Netherlands; 2 Department of Ophthalmology, VU University Medical Centre, Amsterdam, The Netherlands; 3 UCL Institute of Neurology, Queen Square, London, United Kingdom; Charité University Medicine Berlin, Germany

## Abstract

**Purpose:**

Optical coherence tomography (OCT) allows quantification of the thickness of the retinal nerve fibre layer (RNFL) thickness, a potential biomarker for neurodegeneration. The estimated annual RNFL loss in multiple sclerosis amounts to 2 *μ*m using time domain OCT. The recognition of measurement artifacts exceeding this limit is relevant for the successful use of OCT as a secondary outcome measure in clinical trials.

**Methods:**

Prospective study design. An exploratory pilot study (ring and volume scans) followed by a cohort study (1,980 OCT ring scans). The OCT measurement beam was placed off–axis to the left, right, top and bottom of the subjects pupil and RNFL thickness of these scans were compared to the centrally placed reference scans.

**Results:**

Off–axis placement of the OCT measurement beam resulted in significant artifacts in RNFL thickness measurements (95%CI 9*μ*m, maximal size of error 42*μ*m). Off–axis placement gave characteristic patterns of the OCT live images which are not necessarily saved for review. Off–axis placement also causes regional inhomogeneity of reflectivity in the outer nuclear (ONL) and outer plexiform layers (OPL) which remains visible on scans saved for review.

**Conclusion:**

Off–axis beam placement introduces measurement artifacts at a magnitude which may mask recognition of RNFL loss due to neurodegeneration in multiple sclerosis. The resulting pattern in the OCT live image can only be recognised by the technician capturing the scans. Once the averaged scans have been aligned this pattern is lost. Retrospective identification of this artifact is however possible by presence of regional inhomogeneity of ONL/OPL reflectivity. This simple and robust sign may be considered for quality control criteria in the setting of multicentre OCT studies. The practical advice of this study is to keep the OCT image in the acquisition window horizontally aligned whenever possible.

## Introduction

Accurate assessment of neurodegeneration is important for prognosis and evaluation of neuroprotective treatment strategies. Spectral–domain (SD) optical coherence tomography (OCT) allows to quantify RNFL thickness changes with a precision in the range of 1.14–2.39 *μ*m [1]. It has been proposed that OCT measurements of the retina may provide promising primary outcome measures for neuroprotective treatment trials in multiple sclerosis (MS) [2], [3].

The estimated annual loss of RNFL thickness in MS is about 1–2 *μ*m [4] Longitudinal, observational studies are underway to validate these findings with the newer, high resolution SD–OCT technology. As with other imaging studies of neurodegeneration, [2] qualified assessment of the OCT will become a requirement for high quality multicentre studies. At present there are no validated reading centre criteria available for the assessment of OCT scans in MS. A review of the literature shows that the most frequently reported errors are related to boundary line errors, poor signal strength or bad placement of the ring scan at the optic nerve head (ONH) [5] To the best of our knowledge, the potential artifact introduced by off–axis placement of the measurement beam is not known and is therefore investigated in this study.

## Methods

This study was approved by the medical ethical committee (protocol number 2010/336) and the scientific research committee (protocol number CWO/10-22E) of the of the VU University Medical Centre in Amsterdam, the Netherlands. The individuals in the Video S1 have given written informed consent (as outlined in PLOS consent form) to publish the video material.

This study consists of a video documented pilot and a main study. In both studies all scans were recorded by one qualified operator, using SD–OCT (Heidelberg Spectralis, Software version 1.1.6.3) with the eye tracking function enabled. In the pilot study, all scans were obtained in the right eye (0 dpt, both with dilated (Tropicamide 0.5%) and undilated pupil) of one subject. Four OCT scans were performed: (1) a ring scan (diameter 12° or 2.4 cm, 20–25 ART) at the ONH, (2) a macular volume scan (20×20°, 49 ART, 25 sections), (3) a papillomacular bundle (PMB) volume scan set at a 7° angle at the macular of 20° length and 4° width (105 sections) and (4) an ONH volume scan (15×15°, 24 ART, 73 sections). The first of each scan was set as reference. The automated follow–up option was used for repeat scans. The OCT measurement beam was placed about 2 mm off–axis temporal, nasal, superior and inferior from the subjects pupil centre. Locations were changed randomly. A total of 10 measurements were taken per location.

For the main study, all measurements were taken in a dimmed room and pupil size was measured. Pharmacological pupil dilatation was not performed. The same ring scan at the ONH was assessed in both eyes of 11 subjects at 9 locations. The first location was with central beam placement and defined as the reference scan. As for the pilot study, the eye–tracking function was enabled and the very first scan used as reference for automated placement of the ring-scan for the following scans. The second location was a small degree of superior off–axis placement (about 1/3 of the individual pupil size), resulting in a live image slightly deviating from a straight horizontal OCT image. The third location was a larger (about 2/3 of the individual pupil size) superior displacement. The fourth and fifth location were respective small and large temporal displacements. The sixth and seventh location were respective small and large inferior displacements. The eight and ninth location were respective small and large nasal displacements. In addition, all subjects underwent formal automated perimetry using 30–2 threshold test (SITA-Standard strategy) on the Humphrey field analyser (Carl Zeiss Meditec, Dublin, CA, USA). Refractive errors were corrected using wide angle lenses. Visual field data was reported as the overall field mean deviation as derived from control data provided by the manufacturer.

All statistical analyses were performed using SAS software (V9.2). The mean ± standard deviation (SD) are presented. The Kruskal–Wallis test was used for comparison of multiple groups within each retinal sector. Two types of analyses were performed. First, the absolute values of the RNFL thickness were compared for each eye separately in each subject in order to test whether off–axis beam placement may matter on an individual subject basis. Second, a group comparison was performed. In order to compare the RNFL values the absolute size of the error in *μ*m was calculated. Because equal sized errors in different directions (e.g. +x *μ*m and -x *μ*m) average each other statistically, |*x*| was used to indicate the size of the measurement error for descriptive data analysis.

## Results

### Pilot study

The video (Video S1, supplementary data) gives a live coverage of the effect of off-axis measurement beam placement.

**Figure 1:**
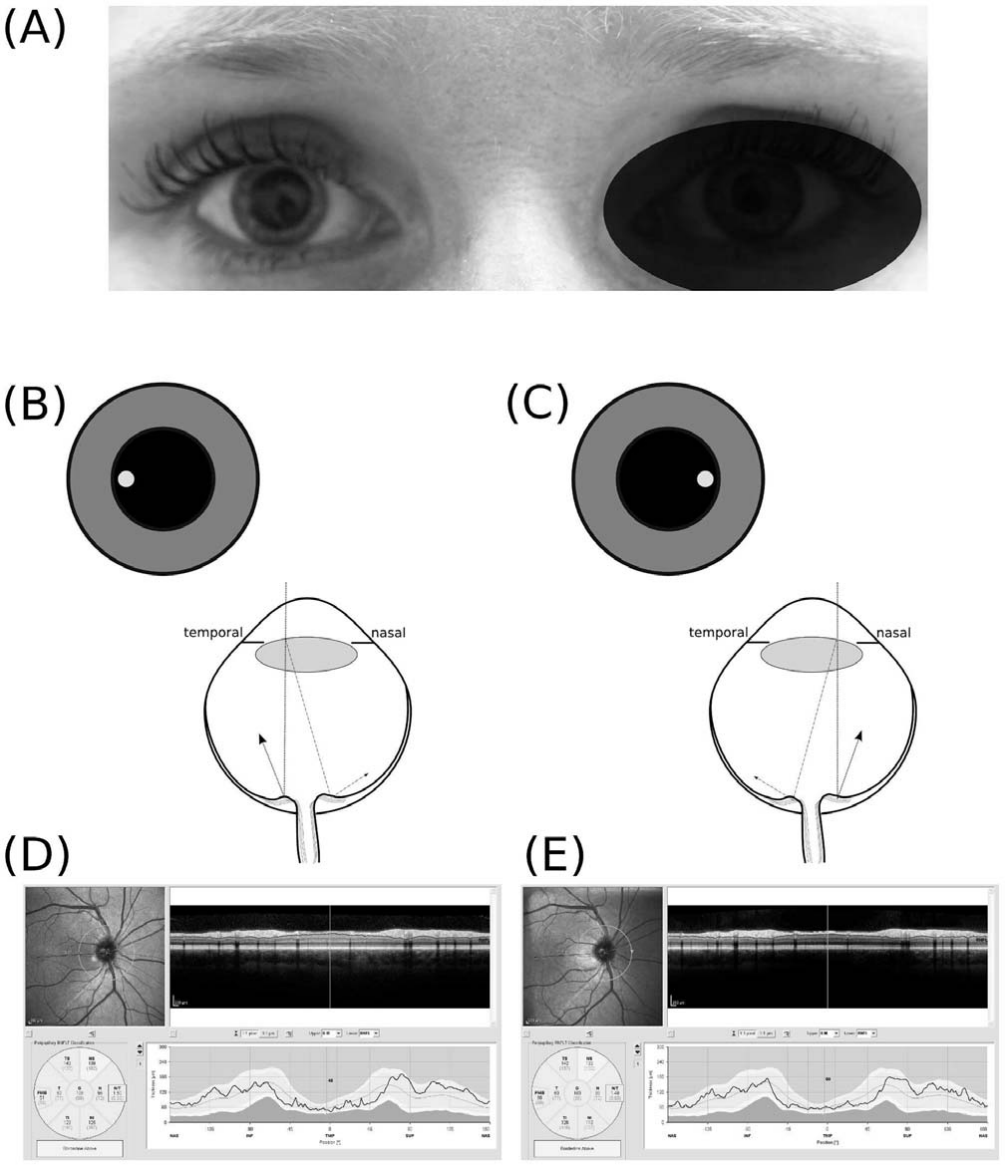
Off centre placement of the measurement beam. (**A**) The OCT measurement beam is focused on the dilated right eye of subject #1, (**B**) temporal off–axis placement of the measurement beam results in a shorter light path to the temporal part of the optic nerve head (dotted line) and a longer pathway from the nasal part of the optic nerve head (dotted-dashed line). The difference in path length results in a tilted appearance of the B–scan. (**C**) Nasal off–axis placement of themeasurement beam results in a mirror pattern. The resulting averaged OCT image is of good quality (ART 25, signal strength 35 dB) for both (**D**) temporal off–axis placement and (**E**) nasal off–axis placement. The quantification of the RNFL thickness by the algorithm is however clearly different (Global average OD with temporal off-axis placement 106 μm and nasal off–axis placement 103 μm).

To illustrate the problem the effect of nasal and temporal off–axis measurement beam placement on RNFL thickness data is shown (Figure 1 A–E). With temporal/nasal off-axis placement the path-length for light reflected from the nasal and temporal proportion of the ONH differ such that the OCT live B–scan appears to be tilted in opposite directions (Figure 1 B&C). This tilt is not anymore seen on the averaged summary image (Figure 1 D&E). The resulting measurement artifacts in per sector ranged from −5 *μ*m for the PMB in the temporal sector to +7 *μ*m (nasal sector) (Figure 1 D&E).

**Figure 2:**
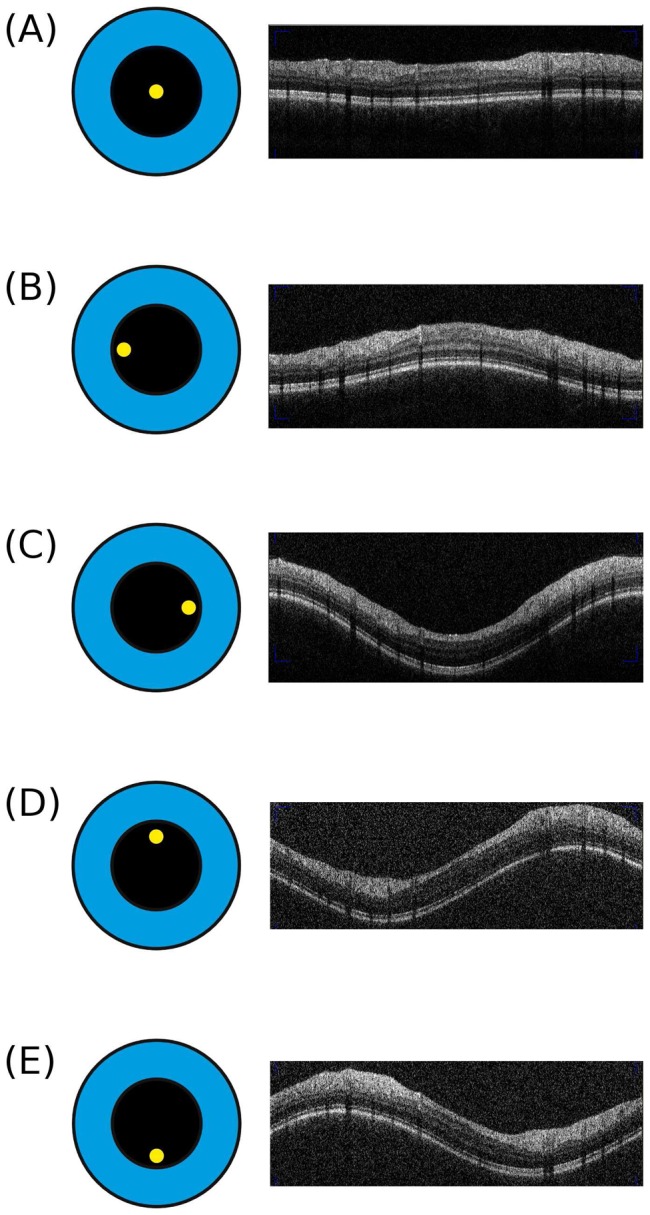
Off centre placement of the OCT measurement beam results in tilted images. Here we show the OCT live image obtained by the optic nerve head ring scan. (**A**) The reference scan with the measurement beam (yellow dot) being placed centrally in the pupil (black circle). This results in a correct, horizontal OCT live image. Note, the live image will not be visible to the reading centre (note the live image was taken as a screen shot during the imaging and appears in print in lower quality than in reality. Please see the video in the supplementary material for a live coverage image acquisition). (**B**) Temporal off–axis placement of the measurement beam results in a centrally convex live image. (**C**) Nasal off–axis placement results in a centrally concave OCT live image. (**D**) Superior off–axis placement results in a rising wave which is mirrored by (**E**) inferior off–axis placement (a falling wave). Please note that for didactic purposes the off–axis placement of the measurement beam is shown for an idealized situation with a central fixation target for a perfectly aligned right subject's eye from the OCT operators point of view.

**Figure 3:**
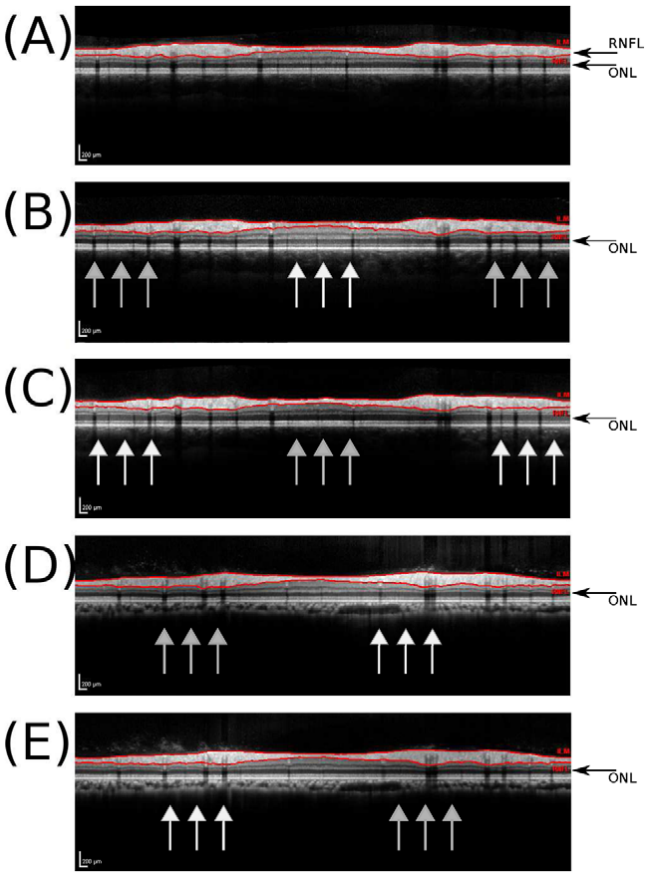
Inhomogeneous reflectivity of the outer part of the ONL indicates off centre placement of the OCT measurement beam. (**A**) The averaged summary scan obtained from the correctly, horizontally orientated live images of the reference scan shown in Figure 2A. This images shows a homogeneous reflectivity of the outer ONL (black arrow). The automated segmentation identifies the borders of the RNFL (red/gray lines). Note, this is the image which is send to the reading centre and used for automated calculation of the RNFL thickness shown in Table 1. (**B**) temporal off-axis placement results in a inhomogeneous outer ONL reflectivity. The ONL reflectivity is increased for the centrally elevated part in Figure 2B (white arrows) and decreased in the periphery (gray arrows). (**C**) nasal off–axis placement (**D**) superior off-axis placement (**E**) inferior off–axis placement.

**Figure 4:**
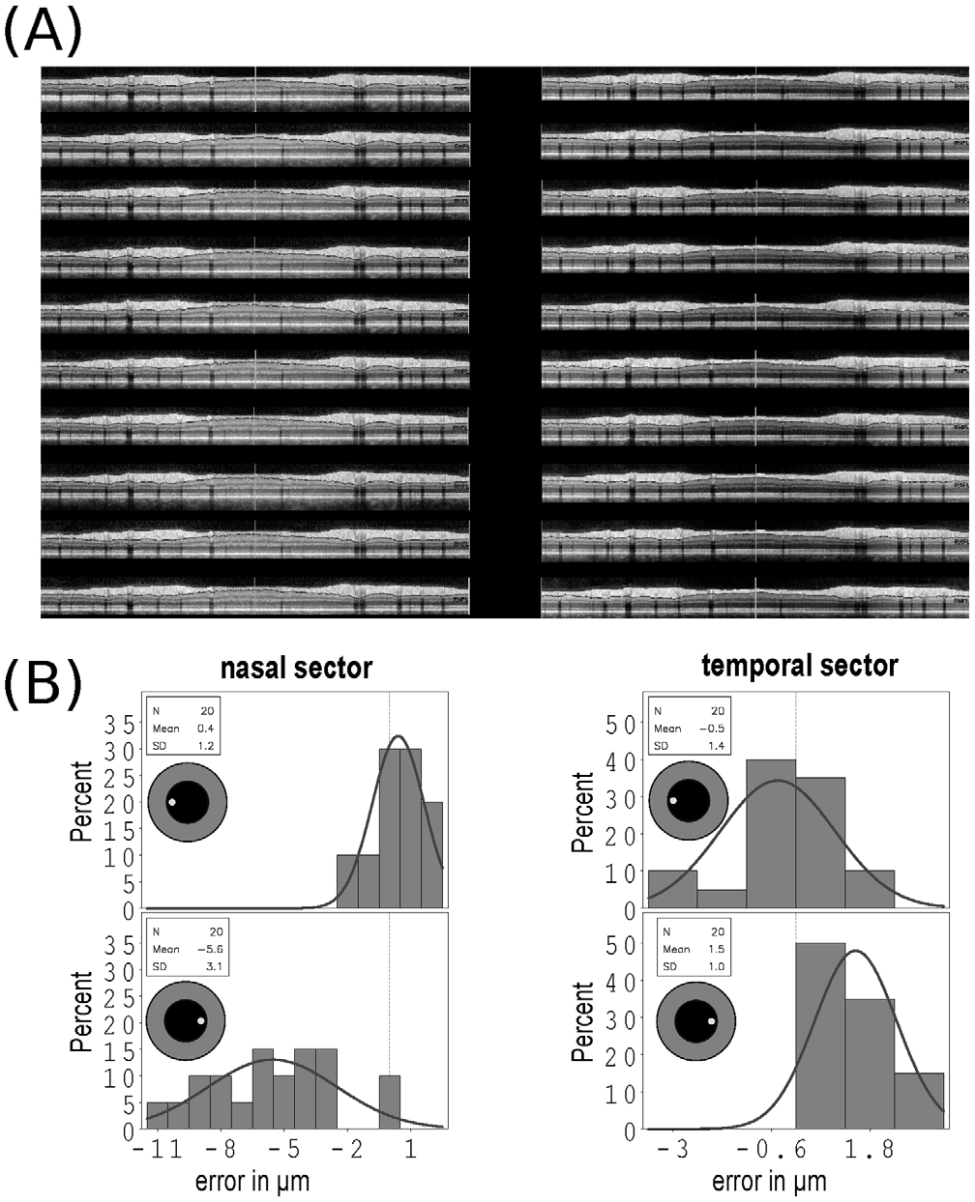
Scan quality and direction of changes in RNFL thickness. (**A**) All averaged ONH ring scan images are of high signal strength and quality (ART 25, signal >35 dB) taken for large temporal and nasal off–axis beam placement from subject #1 (OD) are shown (note the 10 additional scans with only small off-axis placement are of comparable quality). (**B**) The ring scan RNFL data was separately analyzed for the nasal and temporal sectors. The direction and size of the resulting measurement error compared to the reference scan (vertical dashed reference line) are shown as a histogram for the nasal and temporal sectors. The inlay indicates direction of off-axis beam placement in each case. The overlaid Gaussian curve illustrates the mirror pattern of the resulting over-/underestimation of the RNFL thickness in this subject. The y-axis gives the percentage of scans for the range of measurement error in µm shown on the x-axis.

The appearance of the tilted live OCT B–scan is highly reproducible on repeat assessments. A central OCT measurement beam placement always resulted in a horizontally aligned OCT retinal live image (Figure 2 A). Off–axis placement of the OCT measurement beam caused a reproducible and characteristic retinal pattern: (1) centrally convex if placed temporal (Figure 2 B), (2) centrally concave if placed nasal (Figure 2 C), (3) a rising wave if placed superior (Figure 2 D) and (4) a falling wave if placed inferior to the pupil centre (Figure 2 E). Of note, even a relative small degree off–axis beam placement as used for the pilot study will cause a clearly visible wave OCT image waveform on the live screen (see also Video S1).

Importantly, the live image will not be visible to a reading centre. In fact, the images shown in Figure 2 were taken with the computer's screen-shot function. A reading centre will receive a horizontally aligned average image. The average images corresponding to the live images from Figure 2 are shown in Figure 3. With central placement of the measurement beam the ONL reflectivity is homogeneous (bottom black arrow in Figure 3 A). The OCT signal strength was good and the automated segmentation algorithm correctly identifies the RNFL boundaries (red lines in Figure 3 A). Off–axis placement of the OCT measurement beam produces a reproducible change in light backscattering from the ONL. Figure 3 shows the effects on the ONL reflectivity for temporal, nasal, superior and inferior of off–axis placement. This inhomogeneity of the ONL reflectivity was also visible on all volume scans.

Automated, quantitative analysis of the RNFL thickness in the pilot study changed significantly with off–axis placement of the measurement beam. Table 1 shows the results of the scans performed in a undilated pupil. Importantly, the observed artifacts were not different when scans were made with a dilated pupil (data not shown). The largest artifacts (over 10 *μ*m) were observed in the temporal superior (135±17.8 *μ*m versus 148±5.4 *μ*m) and nasal superior sectors (132±23.7 *μ*m versus 143±2.5 *μ*m). This was followed by the PMB (48±0.8 *μ*m versus 61±36.6 *μ*m) and mid temporal sector (60±0.7 *μ*m versus 72±36.4 *μ*m). Measurement artifacts for the mid and inferior nasal, inferior temporal and global mean were less marked, but remained significant (Table 1). Importantly, signal strength was excellent (*>*35 dB) for all scans and there was no algorithm failure accounting for erroneous RNFL thickness measurements (Figure 4 A). Typically, for the nasal sector the RNFL thickness increased with temporal off-axis beam placement and decreased with nasal off–axis beam placement (Figure 4 B). The opposite was observed for the temporal sector.

**Table 1:** Quantification of RNFL thickness.

	Beam placement					
	Central	Right	Left	Top	Bottom	p-value
Global mean [µm]	105±0.6	107±1.2	105±0.8	107±1.3	109±8.6	0.002
PMB [µm]	48±0.8	51±1.5	50±1.0	50±1.8	61±36.6	0.003
Sup. nasal [µm]	137±1.6	143±2.5	137±2.3	142±2.9	132±23.7	<0.0001
Nasal [µm]	98±1.3	95±4.1	97±1.0	100±1.9	98±1.1	0.002
Inf. nasal [µm]	124±1.3	122±1.5	123±1.8	121±1.4	122±1.6	0.03
Inf temporal [µm]	126±1.0	126±1.9	125±1.8	124±1.5	127±1.4	0.01
Temporal [µm]	60±0.7	61±1.1	61±0.7	62±1.6	72±36.4	0.006
Sup. temporal [µm]	140±1.3	148±5.4	142±1.6	144±2.4	135±17.8	<0.0001

The pilot study shows that quantification of the RNFL thickness depends on placement of the measurement beam. The mean±standard deviation are shown. Group comparisons were done using the Kruskal–Wallis test.

PMB =  papillomacular bundle.

### Ring scan main study

A total of 1,980 OCT ring scans were taken from 11 subjects (90 scans per subject per eye). Three scans from subject #8 were rejected because of an algorithm failure. The remaining 1,977 OCT ring scans were used for statistical analyses. The averaged scan quality was 27.4 dB with an ART of 69. The demographic data and average global RNFL thickness per subject and eye are summarized in Table 2. All subjects had normal visual fields on automated perimetry.

**Table 2:** Subject characteristics.

Subject	Age [years]	Sex	Height [cm]	Weight [kg]	Pupil [mm]		Refraction [Dpt]		VF [MD]		RNFL [μm]	
					OD	OS	OD	OS	OD	OS	OD	OS
												
# 1	24	f	177	64	5.0	4.5	0	0	+0.82	+0.73	106	109
# 2	62	f	175	70	5.9	4.8	−1.75	−0.75	+0.12	−0.22	89	93
# 3	27	f	170	63	4.9	4.9	0	−1	−0.77	−1.11	103	106
# 4	27	f	167	91	4.1	3.8	−4	−4	−0.93	−1.01	100	103
# 5	51	m	178	68	3.8	3.9	−4.75	−1.5	+0.56	−0.42	85	86
# 6	49	m	182	90	3.7	3.6	+1	+1	−0.26	−0.73	98	94
# 7	51	f	163	61	5.0	5.5	0	0	+2.18	+1.14	92	92
# 8	31	f	181	74	4.7	4.3	0	0	+1.86	+1.39	96	105
# 9	27	f	176	78	4.5	3.7	−1.5	−1.5	−1.49	−1.08	104	110
# 10	29	f	169	58	4.6	4.8	−2.25	−3.5	−0.52	−0.32	94	93
# 11	24	m	186	75	5.2	4.8	0	0	+0.31	+1.05	98	98

Female  =  f, male  = m, MD  =  mean field deviation, VF  =  visual field.

**Figure 5:**
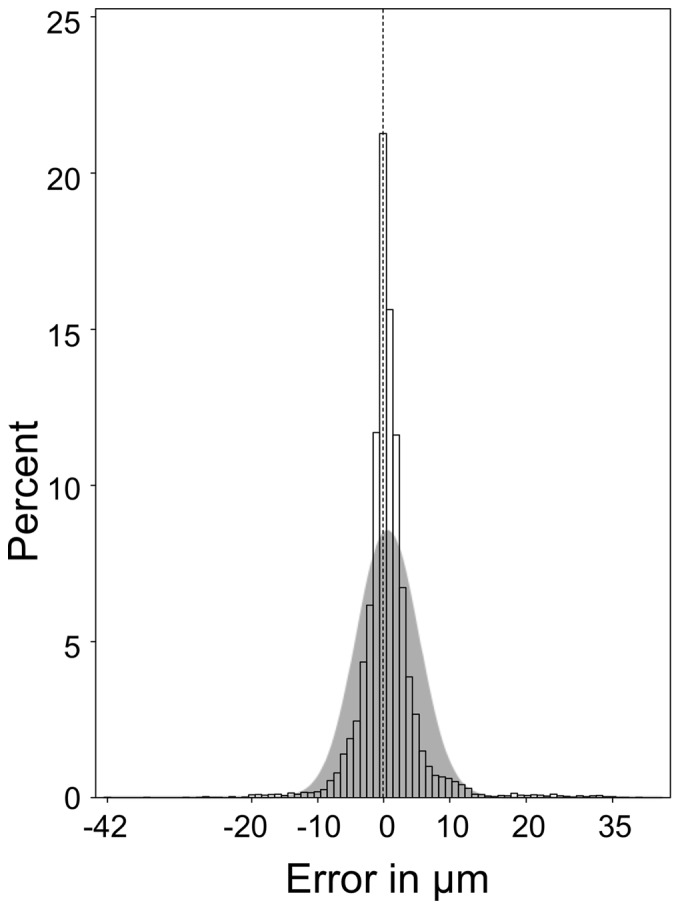
Measurement error in μm caused by off–axis beam placement. In about 78% of all measurements there is an error of ≥|0| μm, in 5% ≥|9| μm with a maximum error of |42| μm. An accurate measurement (0 μm error) is indicated by the dashed vertical reference line. The size of the measurement error demonstrates a Gaussian distribution to both sides of the vertical reference line (gray shaded curve). The y-axis gives the percentage of scans for the range of measurement error in µm shown on the x-axis.

As in the pilot study, measurements done with central beam placement were taken as reference. The *averaged* measurement artifact caused by a small and large degree off-centre beam placement are shown in Table 3. Consistently, a larger off-centre beam placement caused a larger sized measurement error. In the pooled data analysis the size of the measurement artifact was *maximal* 17 *μ*m for the global average RNFL thickness, 20 *μ*m for the entire nasal sector, 31 *μ*m for the superior nasal sector, 37 *μ*m for the inferior nasal sector, 21 *μ*m for the entire temporal sector, 42 *μ*m for the inferior temporal sector, 23 *μm* for the superior temporal sector and 35 *μ*m for the PMB. Importantly, already minimal off–axis placement of the laser beam, with respect to the individual pupil size, gave rise to consistent, significant RNFL thickness measurement artifacts. The distribution of the size of the absolute error (|*x*|) for all measurements from all sectors of all subjects pooled (n = 14,055) appears to be Gaussian (Figure 5).

**Table 3:** The mean measurement artifacts caused by small and large off-centre beam placement compared to central beam placement are shown.

	Right	Left	Top	Bottom
*Small off-centre beam placement*				
Global mean [µm]	0.76 (±0.3)	0.95 (±0.8)	0.83 (±0.5)	0.84 (±0.5)
PMB [µm]	1.51 (±1.0)	1.52 (±0.8)	1.04 (0.9)	1.15 (±0.7)
Sup. nasal [µm]	1.57 (±0.8)	2.06 (±1.7)	1.84 (±1.4)	2.01 (±1.2)
Nasal [µm]	1.68 (±0.8)	2.16 (±2.7)	0.94 (±0.7)	1.99 (±1.0)
Inf. Nasal [µm]	1.83 (±1.2)	2.68 (±2.7)	1.32 (±0.8)	2.57 (±0.9)
Inf. Temporal [µm]	1.62 (±1.2)	2.44 (±2.4)	3.12 (±4.3)	2.20 (±1.1)
Temporal [µm]	0.85 (±0.7)	1.23 (±0.9)	0.82 (±0.6)	1.06 (±0.7)
Sup. temporal [µm]	1.51 (±0.6)	2.11 (±1.8)	1.54 (±0.8)	2.05 (±2.5)
*Large off-centre beam placement*				
Global mean [µm]	0.99 (±0.4)	0.86 (±0.8)	1.02 (±0.8)	1.07 (±0.7)
PMB [µm]	1.69 (±1.3)	1.75 (±1.0)	2.76 (±5.3)	1.74 (±0.9)
Sup. nasal [µm]	1.34 (±0.9)	2.30 (±1.5)	2.61 (±1.9)	2.59 (±2.3)
Nasal [µm]	2.65 (±1.6)	2.91 (±2.7)	1.49 (±1.4)	2.62 (±2.4)
Inf. nasal [µm]	2.36 (±1.2)	2.40 (±2.7)	2.88 (±3.2)	3.3 (±2.2)
Inf. temporal [µm]	2.20 (±1.4)	3.31 (±2.4)	3.23 (±3.6)	2.69 (±2.0)
Temporal[µm]	1.10 (±0.8)	1.42 (±0.8)	2.29 (±3.3)	1.68 (±1.0)
Sup. temporal [µm]	2.31 (±1.5)	2.70 (±2.0)	2.54 (±1.3)	2.32 (±2.0)

Values are reported as mean±standard deviation.

PMB =  papillomacular bundle.

Because of phenotype difference of the ONH the size and distribution of the measurement error was different between subjects. Therefore the statistical analyses were repeated for each subject individually (supporting Tables S1 and S2). In each subject large off–axis placement of the laser beam caused a highly significant (p*<*0.0001) RNFL thickness measurement artifacts in almost all sectors.

## Discussion

This study demonstrates that off–axis placement of the OCT measurement beam causes a significant measurement artifact. The size of the error can be as large as 42 *μ*m, but typically remains within ±9 *μ*m (95%CI). The artifact is reproducible on an individual level in all healthy subjects investigated in this study. This error is readily recognised on the live image and technicians capturing the scans should be trained accordingly.

In the context of multicentre studies it is important to note that a central reading centre will not readily be able to recognize off–axis placement of the OCT measurement beam because an averaged summary scan (Figure 3) is sent instead of the live image captured with a screen-shot during the assessment (Figure 2). We therefore describe a new sign, the outer ONL reflectivity which allows for indirect, retrospective assessment of possible off–axis placement of the OCT measurement beam (Figure 2B–E).

We believe recognition of this artifact is relevant for multicentre studies using OCT. If left unrecognized the artifacts may compromise the value of retinal OCT as a primary outcome measure in treatment trials. The artifacts exceeds the estimated annual loss of the global average RNFL thickness in MS (1–2 *μ*m) [4]. There is a considerable degree of inter–individual variation of the ONH. For this reason the pooled group data of this study, although highly significant, is probably less informative than the detailed analyses of each individual subject.

An important practical question is what degree of off–axis laser measurement beam placement is required to cause a significant measurement artifact? In the pilot study we have shown that the moderate degree of displacement (about 2 mm in a 5 mm sized pupil) we have observed in day–to–day practice in our and other centres as well as during OCT training sessions at scientific meetings is sufficient to give rise to a significant measurement artifact. Not surprisingly a larger displacement which may only occasionally occur under difficult image acquisition conditions causes a much larger measurement artifact. The largest measurement error occur when performing the baseline scan at one extreme (for example to the top) and the follow-up scan at the other extreme (to the bottom). Of course if the second scan would be performed at the same degree off-centre placement as the first, only a systematic error would be introduced. The likelihood that an operator remembers the degree of off-centre placement in an individual patient over time is small. Therefore the practical advice for day-to-day practise is simply to try and get a horizontally aligned scan.

Could the error have been caused by an algorithm failure as suggested by Balasubramanian *et al*. [6]? We do not think so for several reasons. First, in the study by Balasubramanian *et al.* the images were taken out of focus (+2 dpt). Second, ART was set to 2. And third, the RNFL thickness was not measured, but the entire retinal thickness [6]. The resulting algorithm failure was caused by a poor signal (*<*10 dB) and occurred at level of Bruch's membrane (see Figure 4 in reference [6]). In contrast, the present study relied on OCT images with, according to the Balasubramanian *et al.* criteria, excellent signal quality (*>*20 dB) with a high ART. This strongly suggests that the measurement artifact introduced by off-axis beam placement was not caused by poor image quality as previously described [6].

Importantly, already a minimal displacement of the measurement beam, which just about gave the impression of a waveform of the OCT live image caused significant measurement artifacts in all subjects in almost all sectors (see supplementary Table S1). We have attempted to find a mathematical expression describing the relationship between the magnitude of the measurement artifact and the amount of off–axis beam placement by measuring the resulting angle of the tilted OCT live image as performed by Lujan *et al* for line scans in the macular region [7]. Because of the large inter–individual variation of the ONH between subjects it was not possible to express such a relationship for ring scans consistently with one mathematical expression. This is important, because in the occasional patient it may not be possible to obtain a perfect, horizontal aligned OCT ring scan due to anatomical reasons. In such a patient an inhomogeneous pattern of OPL/ONL may need to be accepted, but should be reproduced on follow–up scans.

How can the reproducible change in OPL/ONL reflectivity be explained? For the macular region the explanation is straight forward. The inner third of the OPL comprises photoreceptor synapses and the outer two thirds consist of obliquely orientated axonal extensions, called Henle fibres which are surrounded by the fibres of Müller [8]. In Macaque monkeys, Henle fibres are longest at the macula (300–350 *μ*m) were photoreceptor's and ganglion cells are substantially displaced and shorter (≈12.5 *μ*m) towards the ONH.^8^ The distance between the macula and ONH is about 3.0 mm [9]. Typically, macular Henle fibres are not visible with central placement of the measurement beam [10]. Off–axis placement of the measurement beam results in angled backscattering. Nasal off centre placement causes the OCT measurement beam to be refracted temporally and vice versa. This mechanism is illustrated by the mirror pattern of the live images shown in Figure 2 B&C. The same optic principle applies to off–axis placement to the top and bottom, again revealing mirror pattern images (Figure 2 D&E). Therefore strong backscattering of the Henle fibres results with perpendicular OCT measurement beam placement. In contrast, oblique light backscattering from the Henle fibres results in a reduced outer OPL signal [10]. Although there are similarities between the macaque and human retina, comparable data from humans is to the best of our knowledge not available. It could be that the fibres of Müller and possibly also Müller cells which are present throughout the retina also contribute to the change in signal intensity. In this study the ring scan measured the retina at a radius of 1.2 cm from the ONH centre thus capturing the differently sized Henle fibres [8]. The degree of signal change of backscattered light from the ONL underlying the PMB (long Henle fibres) was more marked than for the other sectors (short Henle fibres) which is what one would expect from the data by Perry and Cowey [8]. We can only speculate that this signal change is due to Henle fibres and the fibres of Müller. An alternative explanation could be an oblique course of other retinal axons originating from the ONL as this layer approaches the human ONH. Acknowledging that we cannot provide a clear-cut anatomical explanation in the absence of histological studies it should be highlighted that this sign is highly reproducible. Future studies are needed to elucidate which degree of change in ONL/OPL reflectivity is relevant in clinical trial practise.

A limitation is that the segmentation software only calculated the RNFL thickness for the ring scan and we have therefore not presented any of the volume data. The change of angled light backscatter from ONL at level of the ONH and from the OPL at level of the macula is however, so consistent that it readily allows for identification of volume scans taken with a off–axis measurement beam. Of note, Lujan *et al.*, using different OCT machines (Cirrus Zeiss and Bioptigen) have demonstrated changes in Henle fibre reflectivity to be associated with macular pathology [7]. Another shortcoming is that we have not tested if this artifact also occurs with other OCT machines. This would need to be tested prospectively. Likewise the sensitivity and specificity of this sign for practice in a reading centre environment will require prospective analysis. Finally, all measurements were taken in healthy eyes and we cannot extrapolate the size of the measurement artifact expected in patients with multiple sclerosis. We would caution against using this sign in ophthalmological diseases which may themselves cause inhomogeneity of the ONL such as central serous retinopathy, non–exudative age–related macular degeneration or drusen [7].

Taken together, this study reports a new sign which allows to correctly identify off-axis placement of the OCT beam. Since off–axis placement of the OCT beam resulted in measurement artifacts over 8–times the estimated annual RNFL loss thought be related to neurodegeneration in MS, we believe this sign should be considered in the context of multicentre studies.

## Supporting Information

Table S1 **Small off-axis beam placement.** A small off–axis placement of the measurement beam compared to central beam placement causes a significant measurement artifact in each eye of all subjects on an individual level. The p–value (Kruskal–Wallis test) for each sector is shown as ns = not significant, p<0.0001 = ***, p<0.001 = **, p<0.01 = *, p<0.05 =  †.
(DOC)

Table S2 **Large off-axis beam placement.** A large off–axis placement of the measurement beam compared to central beam placement causes a significant measurement artifact in each eye of all subjects on an individual level. The p–value (Kruskal–Wallis test) for each sector is shown as ns = not significant, p<0.0001 = ***, p<0.001 = **, p<0.01 = *, p<0.05 =  †, ns  =  not significant.
(DOC)

Video S1 **Live coverage of the effect of off axis measurement beam placement.** This video shows the situation of off-centre beam placement, with the live OCT image in the acquisition window. This image shows the artifacts caused by temporal, nasal, inferior and superior off-centre placement of the measurement beam.
(WMV)
