# Supplementary material for: A Simple Sign for Recognizing Off–Axis OCT Measurement Beam Placement in the Context of Multicentre Studies
Source: PLoS One. 2012 Nov 8;7(11):e48222. doi: 10.1371/journal.pone.0048222 (PMC3493550; doi:10.1371/journal.pone.0048222)
Supplement: Table S1 — Small off-axis beam placement. A small off–axis placement of the measurement beam compared to central beam placement causes a significant measurement artifact in each eye of all subjects on an individual level. The p–value (Kruskal–Wallis test) for each sector is shown as ns = not significant, p<0.0001 = ***, p<0.001 = **, p<0.01 = *, p<0.05 = †. (DOC) [file pone.0048222.s001.doc]

| Subject | eye | PMB | Sup-nas | Nas | Inf-nas | Inf-temp | Temp | Sup-temp | Global |
| --- | --- | --- | --- | --- | --- | --- | --- | --- | --- |
|  |  |  |  |  |  |  |  |  |  |
| # 1 | OD | ns | *** | *** | *** | *** | ** | *** | $\dagger$ |
| # 1 | OS | *** | *** | *** | *** | $\dagger$ | ns | $\dagger$ | *** |
| # 2 | OD | *** | *** | * | ** | $\dagger$ | *** | *** | *** |
| # 2 | OS | *** | $\dagger$ | *** | *** | ** | *** | ns | *** |
| # 3 | OD | $\dagger$ | *** | *** | *** | *** | * | *** | *** |
| # 3 | OS | *** | *** | *** | ns | *** | *** | ** | *** |
| # 4 | OD | *** | *** | *** | *** | *** | *** | *** | *** |
| # 4 | OS | *** | *** | *** | ns | *** | *** | *** | *** |
| # 5 | OD | *** | *** | ** | $\dagger$ | ** | *** | *** | ** |
| # 5 | OS | *** | *** | *** | $\dagger$ | *** | *** | *** | ns |
| # 6 | OD | *** | *** | *** | *** | *** | * | * | *** |
| # 6 | OS | *** | *** | *** | *** | *** | ns | *** | ** |
| # 7 | OD | *** | *** | *** | *** | ** | *** | ** | *** |
| # 7 | OS | *** | *** | *** | *** | ns | ** | $\dagger$ | *** |
| # 8 | OD | $\dagger$ | * | *** | ns | *** | *** | $\dagger$ | * |
| # 8 | OS | *** | *** | *** | *** | *** | *** | *** | *** |
| # 9 | OD | *** | *** | *** | *** | *** | *** | ns | *** |
| # 9 | OS | *** | *** | *** | *** | *** | *** | *** | *** |
| # 10 | OD | *** | *** | *** | *** | *** | *** | *** | *** |
| # 10 | OS | *** | *** | *** | *** | *** | ** | *** | ** |
| # 11 | OD | *** | *** | *** | *** | *** | *** | *** | *** |
| # 11 | OS | *** | *** | ns | *** | * | *** | *** | ns |

Table S1: Small off-axis beam placement

A small off–axis placement of the measurement beam compared to central beam placement causes a significant measurement artifact in each eye of all subjects on an individual level. The p–value (Kruskal–Wallis test) for each sector is shown as ns=not significant, p<0.0001 =***, p<0.001 =**, p<0.01 =*, p<0.05 = †.
